# Improve the dosimetric outcome in bilateral head and neck cancer (HNC) treatment using spot-scanning proton arc (SPArc) therapy: a feasibility study

**DOI:** 10.1186/s13014-020-1476-9

**Published:** 2020-01-30

**Authors:** Gang Liu, Xiaoqiang Li, An Qin, Weili Zheng, Di Yan, Sheng Zhang, Craig Stevens, Peyman Kabolizadeh, Xuanfeng Ding

**Affiliations:** 1 Cancer Center, Union Hospital, Tongji Medical College, Huazhong University of Science and Technology, Wuhan, 430023 China; 2 Department of Radiation Oncology, Beaumont Health System, Royal Oak, MI 48074 USA; 3 School of Physics and Technology, Wuhan University, Hubei, Wuhan, 430072 China

**Keywords:** Dosimetric outcome; bilateral head and neck cancer; spot-scanning; proton arc; delivery efficiency, Robustness

## Abstract

**Background:**

To explore the dosimetric improvement, delivery efficiency, and plan robustness for bilateral head and neck cancer (HNC) treatment utilizing a novel proton therapy technique – the spot-scanning proton arc (SPArc) therapy.

**Methods:**

We evaluated fourteen bilateral HNC patients retrospectively. Both SPArc and 3-field Intensity Modulated Proton Therapy (IMPT) plans were generated for each patient using the same robust optimization parameters. The prescription doses were 70Gy (relative biological effectiveness (RBE) for CTV_high and 60Gy[RBE] for CTV_low. Clinically significant dosimetric parameters were extracted and compared. Root-mean-square deviation dose (RMSDs) Volume Histogram(RVH) was used to evaluate the plan robustness. Total treatment delivery time was estimated based on the machine parameters.

**Results:**

The SPArc plan was able to provide equivalent or better robust target coverage while showed significant dosimetric improvements over IMPT in most of the organs at risk (OARs). More specifically, it reduced the mean dose of the ipsilateral parotid, contralateral parotid, and oral cavity by 25.8%(*p* = 0.001), 20.8%(*p* = 0.001) and 20.3%(p = 0.001) respectively compared to IMPT. This technique reduced D1 (the maximum dose covering 1% volume of a structure) of cord and brain stem by 20.8% (*p* = 0.009) and 10.7% (*p* = 0.048), respectively. SPArc also reduced the average integral dose by 17.2%(p = 0.001) and external V3Gy (the volume received 3Gy[RBE]) by 8.3%(*p* = 0.008) as well. RVH analysis showed that the SPArc plans reduced the dose uncertainties in most OARs compared to IMPT, such as cord: 1.1 ± 0.4Gy[RBE] vs 0.7 ± 0.3Gy[RBE](p = 0.001), brain stem: 0.9 ± 0.7Gy[RBE] vs 0.7 ± 0.7Gy[RBE](*p* = 0.019), contralateral parotid: 2.5 ± 0.5Gy[RBE] vs 2.2 ± 0.6Gy[RBE](*p* = 0.022) and ipsilateral parotid: 3.1 ± 0.7Gy[RBE] vs 2.8 ± 0.6Gy[RBE](*p* = 0.004) respectively. The average total estimated treatment delivery time were 283.4 ± 56.2 s, 469.2 ± 62.0 s and 1294.9 ± 106.7 s based on energy-layer-switching-time (ELST) of 0.1 s, 1 s, and 5 s respectively for SPArc plans, compared to the respective values of 328.0 ± 47.6 s(*p* = 0.002), 434.1 ± 52.0 s(p = 0.002), and 901.7 ± 74.8 s(*p* = 0.001) for 3-field IMPT plans. The potential clinical benefit of utilizing SPArc will lead to a decrease in the mean probability of salivary flow dysfunction by 31.3%(p = 0.001) compared with IMPT.

**Conclusions:**

SPArc could significantly spare OARs while providing a similar or better robust target coverage compared with IMPT in the treatment of bilateral HNC. In the modern proton system with ELST less than 0.5 s, SPArc could potentially be implemented in the routine clinic with a practical, achievable treatment delivery efficiency.

## Background

The management of head and neck cancer (HNC) frequently involves radiation treatment. Previous studies have shown that external beam radiation therapy, including 3-dimensional conformal radiation therapy (3DCRT), intensity-modulated photon therapy (IMRT), and volumetric-modulated arc therapy (VMAT), improved HNC patient’s outcome and quality of life significantly [1–3]. Currently, IMRT is the standard of treatment delivery for HNC, given its ability to spare the adjacent organs-at-risk (OARs). On the other hand, proton beam therapy has been introduced to clinical practice to achieve better dose conformity and better OARs sparing such as spinal cord, brain stem, oral cavity, and parotid compared to IMRT or VMAT [4–6].

The recent development of Pencil Beam Scanning (PBS) technique, such as Intensity Modulated Proton Therapy (IMPT), utilizes numerous mono-energetic narrow beamlets (“spots”) to paint the target volume layer by layer. Such a technique significantly improves the dose conformity over the traditional proton passive-scattering technique. Although this active scanning delivery system provides the most considerable flexibility to shape the target volume dose distribution and pattern just like 3D printing in the human body, it is also highly susceptible to different uncertainties, including setup, range uncertainties [7–9], and especially geometric changes in bilateral HNC patients [10].

Due to the current technique limitation of proton treatment delivery efficiency, only a few beam angles are utilized for the treatment of bilateral HNC [11, 12]. Furthermore, the inherent characteristic of increased lateral penumbra due to the scattering may result in undesirable plan quality and conformity, which become very challenging to spare the critical structures adjacent treatment site using IMPT, such as the parotid and oral cavity in HNC [13, 14]. Besides, salivary flow dysfunction and xerostomia are the most common late side effects of radiotherapy (RT) for head-and-neck malignancies and a significant cause of decreased quality of life in survivors [15]. Thus, there is an immediate need to improve further the dosimetric treatment plan quality. A more recent study in 2018 found that with increased degrees of optimization freedom or more beam directions, the range shifter (RS) may not be needed in the treatment of bilateral HNC compared to the current standard-of-care approach, IMPT [12]. The study also found an interesting phenomenon that the dosimetric plan quality is getting better even without utilizing RS in bilateral HNC treatment. Unfortunately, such a proposal and approach with numerous beam angles may not be practically feasible in a proton therapy center due to prolonged treatment time. However, the idea of delivering the proton beam therapy through arc(s) trajectory with a continuously rotational gantry may overcome such limitation [11, 16]. A new treatment technique, Spot-scanning Proton Arc therapy (SPArc) introduced by Ding et al. in 2016 could generate robust and delivery efficient proton arc therapy plans. This technique has the potential to be implemented into the existing clinical proton system [17, 18]. To the best of our knowledge, this is the first comprehensive study to investigate the potential dosimetric and clinical benefits, delivery efficiency, delivery accuracy, and plan robustness of SPArc in the treatment of bilateral HNC.

## Methods

### Treatment planning

Fourteen bilateral HNC patients’ computed tomography (CT) scans were studied in this work. All the patients were simulated and treated in a supine position using customized thermoplastic masks. The target volumes were CTV_high (high risk target volume) and CTV_low (low risk volume, including lymph nodes). OARs considered were parotids, brainstem, cord, and oral cavity. The volumes of the target and OARs were listed in Table 1. Doses were prescribed in Gy relative biological effectiveness [RBE], assuming an RBE value of 1.1 for protons. The prescribed dose to CTV_high was 70Gy [RBE] in 35 fractions of 2Gy [RBE] per fraction; to CTV_low, in 1.71Gy [RBE] per fraction. The planning goals were as follows: 98% of the CTVs had to be covered by 100% of the prescription dose. The similar objectives and constraints for organs OARs were used for both plans.

**Table 1:** The volume of targets and OARs for fourteen patients. (Unit:cc)

NO.	CTV high	CTV low/median	Ipsilateral Parotid	Contralateral Parotid	Cord	Brain Stem	Oral Cavity
1	141.7	204.5	38.8	39.4	16.3	12.9	74.6
2	418.2	202.4	23.9	12.7	18.7	24.6	81.5
3	92.8	172.1	19.8	12.7	16.0	29.2	190.3
4	173.7	400.5	53.1	57.0	12.8	19.8	136.3
5	234.2	240.7	29.8	30.5	33.2	30.6	109.0
6	127.9	283.4	43.0	41.2	8.9	7.9	55.7
7	106.7	119.3	20.1	23.6	25.9	7.1	61.4
8	124.3	324.0	43.7	41.8	14.2	20.8	127.2
9	174.2	394.2	20.2	27.6	49.3	36.7	52.0
10	238.6	200.8	30.2	35.7	19.1	28.0	44.7
11	65.7	374.6	22.4	26.8	17.0	28.9	95.7
12	100.8	286.8	33.8	31.7	29.7	19.4	59.7
13	97.6	166.7	32.2	39.6	30.1	15.3	49.2
14	58.0	225.3	18.9	18.2	31.6	13.8	35.3
Average	153.9	256.8	30.7	31.3	23.1	21.1	83.7
Standard Deviation	94.0	89.4	10.7	12.3	10.8	9.0	43.7

For beam arrangements, three fields (bilateral oblique directions and one Posterior-Anterior direction) were used for IMPT plan. SPArc plan utilized a full arc trajectory (360 degrees) with a sampling frequency of 2.5 degrees pre-control point [17] without using an RS. Both IMPT and SPArc treatment plans were generated using the RayStation (RaySearch Laboratories AB, Stockholm, Sweden) treatment planning system (TPS) version 6.0 with the same robust optimization parameters: ±3.5% range and 3 mm setup uncertainties (a total of 21 worst-case-scenarios), 3 mm dose grid and 0.02 minimum monitor unit (MU) per spot.

### SPArc algorithm

SPArc is an advanced form of IMPT. Its optimization algorithm was based on the worst-case scenario robust optimization integrated with iterative approaches, including (1) control point re-sampling, (2) control point energy layers re-distribution, and (3) control point energy layers filtration, (4) energy layers re-sampling and (5) spot number reduction. With these approaches, SPArc generates plans that are both robust and delivery efficient, which have the potential to be delivered using the existing clinical proton system [18]. Details of the algorithm are described by Ding et al. in 2016 [17].

### Plan quality and robustness evaluation

The DVHs of both target volumes and OARs were generated on the nominal dose distributions for plan quality evaluation. Clinically significant dosimetric parameters including the maximum dose (D1, the maximum dose covering 1% volume of a structure) of the brain stem and spinal cord, mean dose of the parotid and, oral cavity were analyzed. Homogeneity Index (HI) was also evaluated based on the Radiation Therapy Oncology Group (RTOG) recommendations, calculated as follows:

1 $$ HI=\frac{D5}{D95} $$

where D*x* is the maximum dose covering *x*% volume of a structure.

The conformality index (CI) of target coverage was evaluated as [19, 20]:

2 $$ CI=\frac{ TV Dp}{TV}\times \frac{ TV Dp}{VDp} $$

where *TVDp, TV,* and *VDp* are the target volume covered by the prescribed dose, target volume, and the volume enclosed by the prescription isodose line respectively.

The integral dose (ID (Gy · L)) of radiation delivered to the whole patient body structure or external was defined as:

3 $$ \mathrm{ID}=\overline{D}\cdot \mathrm{V}\kern0.5em $$

where $\overline{D}$ (Gy) is the mean dose delivered to volume V (L) (where L – liter). ID formula was employed to calculate and compare the absorbed dose in the patient body [21].

For plan robustness evaluation, the perturbed dose for both SPArc and IMPT plans were generated with 3 mm isocenter shift in the anterior-posterior, superior-inferior, and right-left directions under nominal proton beam range, with + 3.5% and − 3.5% proton beam ranges uncertainties, corresponding to a total of 21 dose distribution scenarios. The DVHs for all the scenarios were plotted for comparisons. The root-mean-square dose (RMSD) for each voxel was calculated as a measure of the robustness. The RMSD volume histograms (RVHs) and the Areas Under the RVH Curve (AUC) were compared to quantitatively evaluate the robustness [11, 20, 22, 23]. The smaller the AUC value indicated better plan robustness.

### Treatment delivery efficiency estimation

For delivery efficiency comparison between SPArc and IMPT, total treatment delivery times were estimated based on a 360 gantry with 1 revolution per minute (RPM) gantry rotation speed, 2 ms spot position switching time, Energy Layer Switching Time (ELST) from 0.1 s to 5 s simulating different proton machines in the current market [17].

### Potential clinical benefit for parotid glands

The benefits of SPArc in the clinical setting were estimated using the normal tissue complication probability (NTCP) model. This model was utilized to predict the probability of a reduction in salivary flow to < 25% of the baseline level at ≤6 months after radiotherapy [24].

We use the NTCP model defined as below [1, 25, 26]:

4 $$ \mathrm{NTCP}=\frac{1}{\sqrt{2\pi }}{\int}_{-\infty}^t{e}^{-\frac{x^2}{2}} dx,t=\frac{\mathrm{MD}-T{D}_{50}}{mT{D}_{50}} $$

Where *MD* is the mean organ dose, *TD*_*50*_ is the uniform dose given to the entire organ volume that results in 50% complication risk, m is a measure of the slope of the sigmoid curve represented by the integral of the normal distribution. ‘*m*’ and ‘*TD*_*50*_’were set as 0.53, 31.4Gy, respectively using combined organ analysis of the Chao et al. data [1]. The mean dose was given to both parotid glands in this study.

### Statistic analysis

The difference of all parameters between IMPT and SPArc was assessed with a paired, 2-tailed non-parametric Wilcoxon signed-rank test using SPSS 21.0 software (International Business Machines, Armonk, New York) and *p* values less than 0.05 were considered statistically significant.

## Results

### Plan Dosimetric comparison

**Fig. 1:**
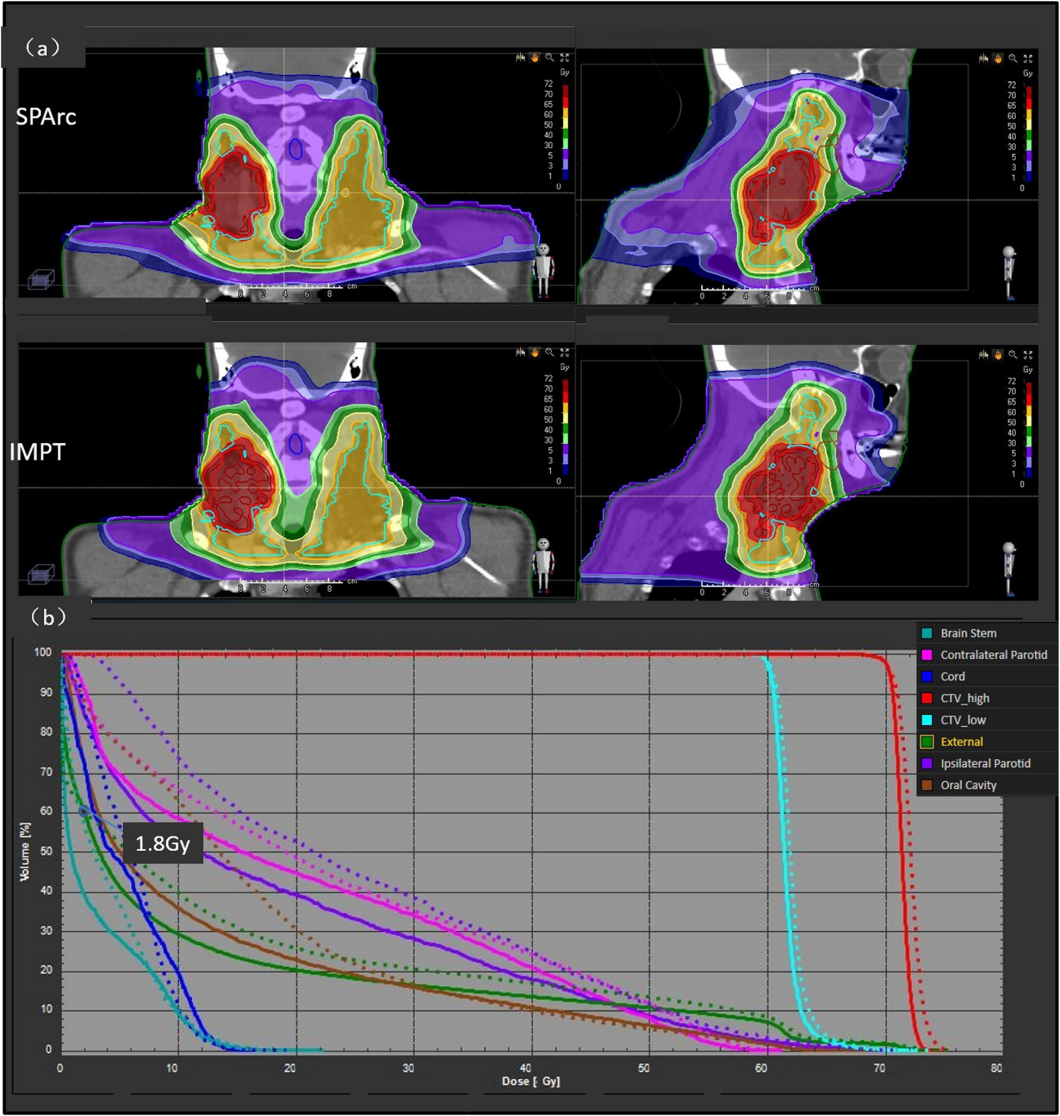
The comparison of (**a**) dose distributions of SPArc and IMPT plans for patient 8 (**b**) the corresponding DVH as an example (SPArc:solid line, IMPT:dash line). The dot as 1.8Gy represents the intersection of external between SPArc and IMPT plan

The study showed that the SPArc treatment plans achieved better conformal dose distribution compared to the IMPT. Figure 1 shows a representative CT slice of a bilateral HNC case (patient #8) with coronal and sagittal view dose comparison for SPArc and IMPT as well as the corresponding DVHs, respectively. The average HI and CI of SPArc plan are 1.04 ± 0.01 and 0.70 ± 0.06 for CTV_high, 1.07 ± 0.04 and 0.54 ± 0.09 for CTV_low, which are significantly improved in comparison with IMPT (CTV_high: HI = 1.05 ± 0.01, *p* = 0.005,CI = 0.67 ± 0.07,*P* = 0.006; CTV_low: HI = 1.09 ± 0.05, *p* = 0.003,CI = 0.51 ± 0.10,*p* = 0.017). Table 2 listed the dosimetric metrics of fourteen patients SPArc and IMPT treatment plans. SPArc had demonstrated significant dosimetric improvements over IMPT throughout all critical organs analyzed. More specifically, SPArc decreased the mean dose to the ipsilateral parotid, contralateral parotid, and oral cavity by 25.8% (*p* = 0.001), 20.8% (p = 0.001) and 20.3%(p = 0.001) respectively. Moreover, the D1 of cord and brain stem was decreased by SPArc (20.8%, *p* = 0.009, and 10.7%, *p* = 0.048). Also, SPArc plans decreased the average integral dose from 147.0 ± 33.8Gy · L to 121.8 ± 29.7Gy · L, by 17.2% (p = 0.001) compared to IMPT plans. SPArc improved the V3Gy of external from 5137.8 ± 1331.7 cc to 4710.8 ± 1328.4 cc(*p* = 0.008) as well.

**Table 2:** The average dosimetric characteristic for the fourteen patients

Structure	Value	SPArc	IMPT	*p*Value
CTV_high	D95(Gy) [RBE]	70.8 ± 0.2	70.7 ± 0.2	0.975
	D5(Gy) [RBE]	73.6 ± 0.8	74.1 ± 0.7	0.011
	HI	1.04 ± 0.01	1.05 ± 0.01	0.005
	CI	0.70 ± 0.06	0.67 ± 0.07	0.006
CTV_low	D95(Gy) [RBE]	60.8 ± 0.4	60.8 ± 0.4	0.777
	D5(Gy) [RBE]	64.6 ± 1.0	65.7 ± 1.7	0.003
	HI	1.07 ± 0.04	1.09 ± 0.05	0.003
	CI	0.54 ± 0.09	0.51 ± 0.10	0.017
Cord	D1(Gy) [RBE]	17.2 ± 7.0	21.7 ± 7.9	0.009
Brain Stem	D1(Gy) [RBE]	14.2 ± 11.1	15.9 ± 11.3	0.048
Contralateral Parotid	Mean Dose (Gy) [RBE]	17.9 ± 6.4	22.6 ± 5.3	0.001
Ipsilateral Parotid	Mean Dose (Gy) [RBE]	21.1 ± 7.4	28.4 ± 6.2	0.001
Oral Cavity	Mean Dose (Gy) [RBE]	21.9 ± 9.7	27.5 ± 9.4	0.001
External	ID (Gy · L)	121.8 ± 29.7	147.0 ± 33.8	0.001
V1Gy	cc	6186.9 ± 1800.1	5852.7 ± 1543.5	0.011
V3Gy	cc	4710.8 ± 1328.4	5137.8 ± 1331.7	0.008

*Abbreviations: HI* (homogeneity index), *RBE* relative biological effectiveness, *Dx* the maximum dose covering *x*% volume of a structure, *ID* the integral dose, *V*_*x*_ the volume of a structure received *x* Gy [RBE]

### Plan robustness evaluation

**Fig. 2:**
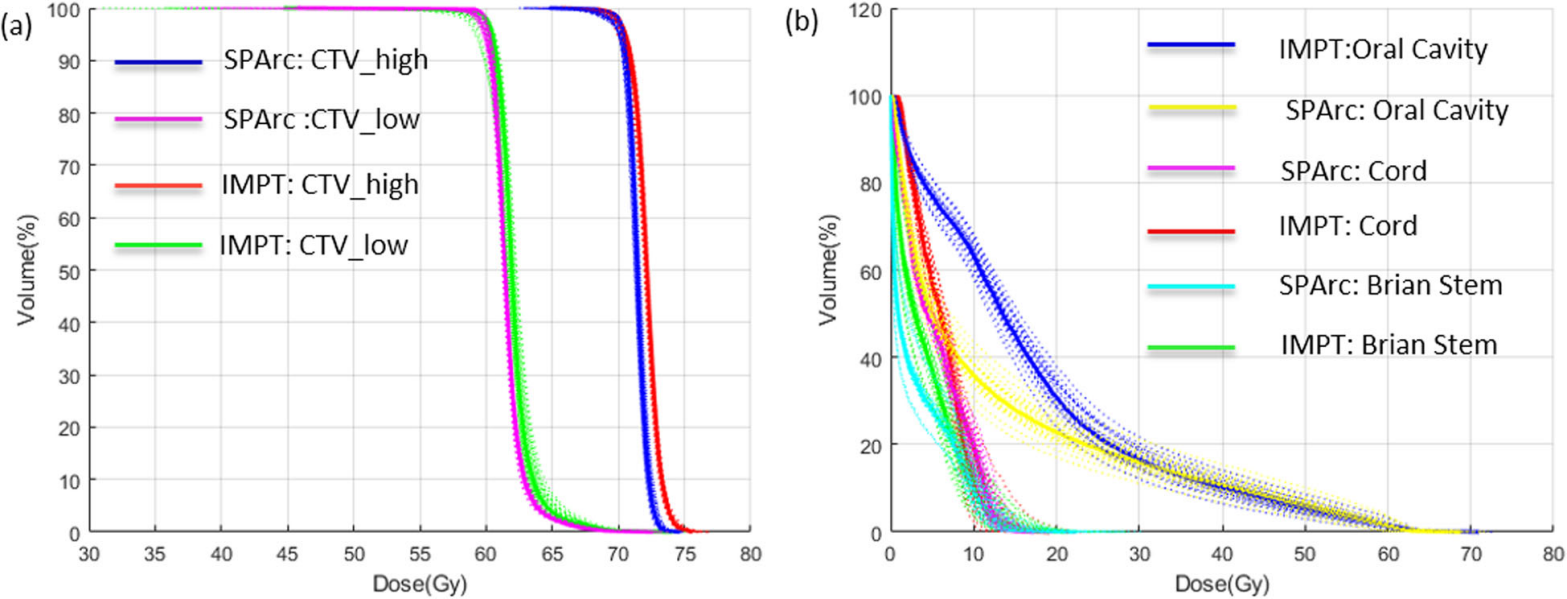
The DVHs of nominal position (solid line) and 20 scenarios of uncertainties (dashed line) for (**a**) target and (**b**) OARs for patient #8

**Fig. 3:**
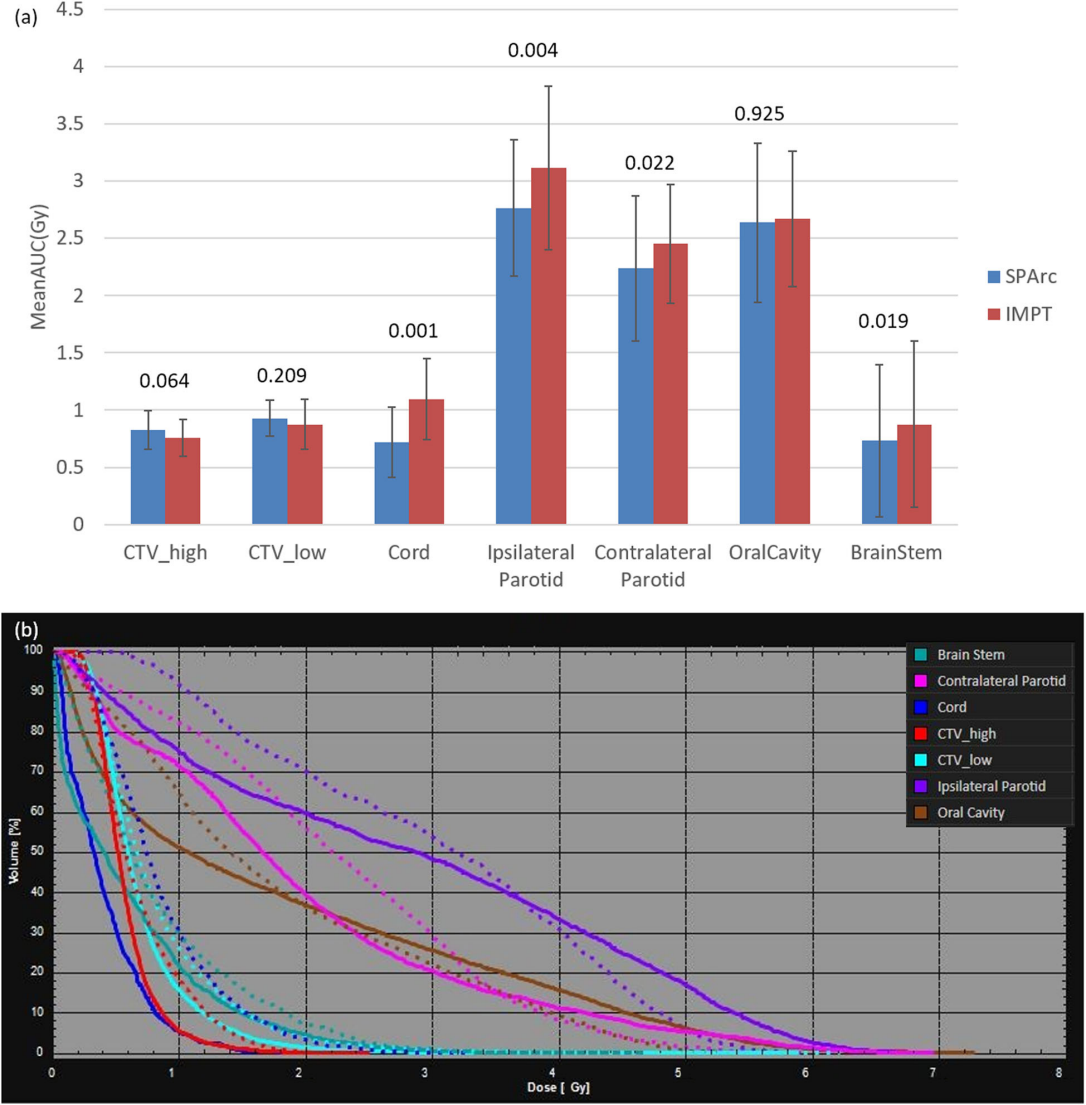
**a** The average areas under the RVH curve (AUC) for fourteen patients with *p*-values on the top of the columns. **b** An example of the root-mean-square dose (RMSD) volume histograms of SPArc (solid line) and IMPT (dashed line) for patient # 8

The robustness of all the treatment plans was evaluated with 21 worst-case-scenarios. The perturbed DVHs for patient #8 are shown in Fig. 2 (a) target coverage, and Fig. 2 (b) OARs sparing. Both SPArc and IMPT plans could achieve adequate target coverage, with at least 98% of CTV_high receiving the prescription dose. Compared to IMPT, the SPArc plan significantly reduced the average AUC value for the cord from 1.1 ± 0.4Gy [RBE] to 0.7 ± 0.3Gy [RBE] (p = 0.001), for brain stem from 0.9 ± 0.7Gy[RBE] to 0.7 ± 0.7Gy[RBE] (*p* = 0.019), for contralateral parotid from 2.5 ± 0.5 cGy [RBE] to 2.2 ± 0.6Gy [RBE] (*p* = 0.022) and for ipsilateral parotid from 3.1 ± 0.7 cGy [RBE] to 2.8 ± 0.6Gy[RBE] (*p* = 0.004). SPArc treatment plan is comparable to IMPT plan in terms of robustness for CTV_high, CTV_low, and oral cavity. Moreover, Fig. 3 (a) showed the corresponding average mean AUC index of target volumes and OARs of the fourteen cases with their corresponding p values. Moreover, an example of the root-mean-square dose (RMSD) volume histograms of SPArc (solid line) and IMPT for patient #8 is given in Fig. 3 (b).

### Total estimated treatment delivery time

**Fig. 4:**
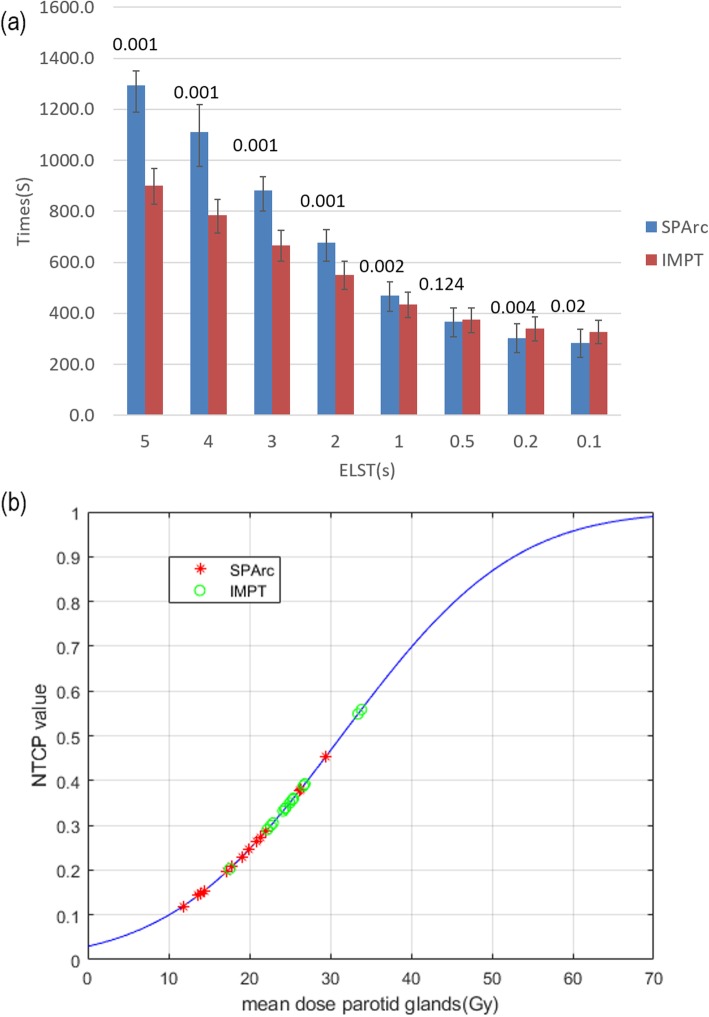
**a** The average estimated delivery time per fraction varies along with the ELST with p-values on the top of the columns. **b** Normal tissue complication probability (NTCP) model for parotid salivary flow (solid curve). The NTCP value denotes the probability of a reduction in salivary flow to < 25% of the pretreatment flow at ≤6 months after radiotherapy

Figure 4(a) shows the estimated delivery time per fraction for both SPArc and IMPT plans simulating proton systems with various ELST. For the proton centers with ELST equaling to 5 s, the estimated delivery time is significantly longer for SPArc (1294.9 ± 106.7 s vs. 901.7 ± 74.8 s *p* < 0.001), however, the difference of treatment delivery time decreases rapidly as the ELST shortens. The delivery time for both modalities are comparable at the ELST = 0.5 s, 366.0 ± 58.5 s(SPArc) vs374.6 ± 49.7 s(IMPT) (*p* = 0.124), and it is even significantly shorter in SPArc plan at ELST≤0.2 s, 303.3 ± 57.3 s(SPArc) vs 339.7 ± 48.1 s (IMPT) (p = 0.004) with ELST = 0.2 s, 283.4 ± 56.2 s(SPArc) vs 328.0 ± 47.6 s(IMPT) (*p* = 0.002) with ELST = 0.1 s.

The average calculation time required to generate a SPArc plan for bilateral HNC is between 6 to 8 h, depending on the size of the target volume. This simulation was performed on a 64-bit workstation with an Intel Quad-Core processor (TM i5–4590 CPU @ 3.30 GHz) and 64 GB RAM.

### Potential clinical benefit for parotid glands

The results showed that there was a potential clinical benefit in terms of estimated salivary flow dysfunction based on mean NTCP values while comparing SPArc to IMPT. The estimated reduction in NTCP varied widely among patients (Fig. 4(b)). As compared with IMPT, SPArc decreased the mean probability of salivary flow dysfunction from 36 ± 9 to 25% ± 10%(p = 0.001) after radiotherapy.

## Discussion

Radiation-induced side effects, including xerostomia and dysphagia, have significant impacts on the quality of life of HNC patients [27–29]. Dosimetric improvements could potentially improve the treatment outcome by reducing acute and late toxicities. Recent studies have shown improved IMPT robustness associated with the increase of treatment fields while decreasing OARs sparing [12, 30, 31]. However, such approaches using numerous static beam angles were considered impractical due to the prolonged treatment delivery time, especially in a multi-room proton center where significant time will be spent in the room-switching time/waiting time [32]. Since the introduction of the SPArc technique in 2016 [17], there have been efforts to investigate the potential dosimetric improvements via SPArc in different disease sites [11, 20, 23]. Our work is the first comprehensive study to exploit the potential advantage of utilizing SPArc for bilateral HNC radiotherapy in terms of OARs sparing, target robust coverage, and treatment delivery efficiency. These results not only consolidated the findings from previous publications regarding further improving the plan quality [33, 34] but also introduced a novel treatment technique that could shorten the proton treatment time and simplify the proton clinical treatment workflow without using an RS through a dynamic arc trajectory.

**Fig. 5:**
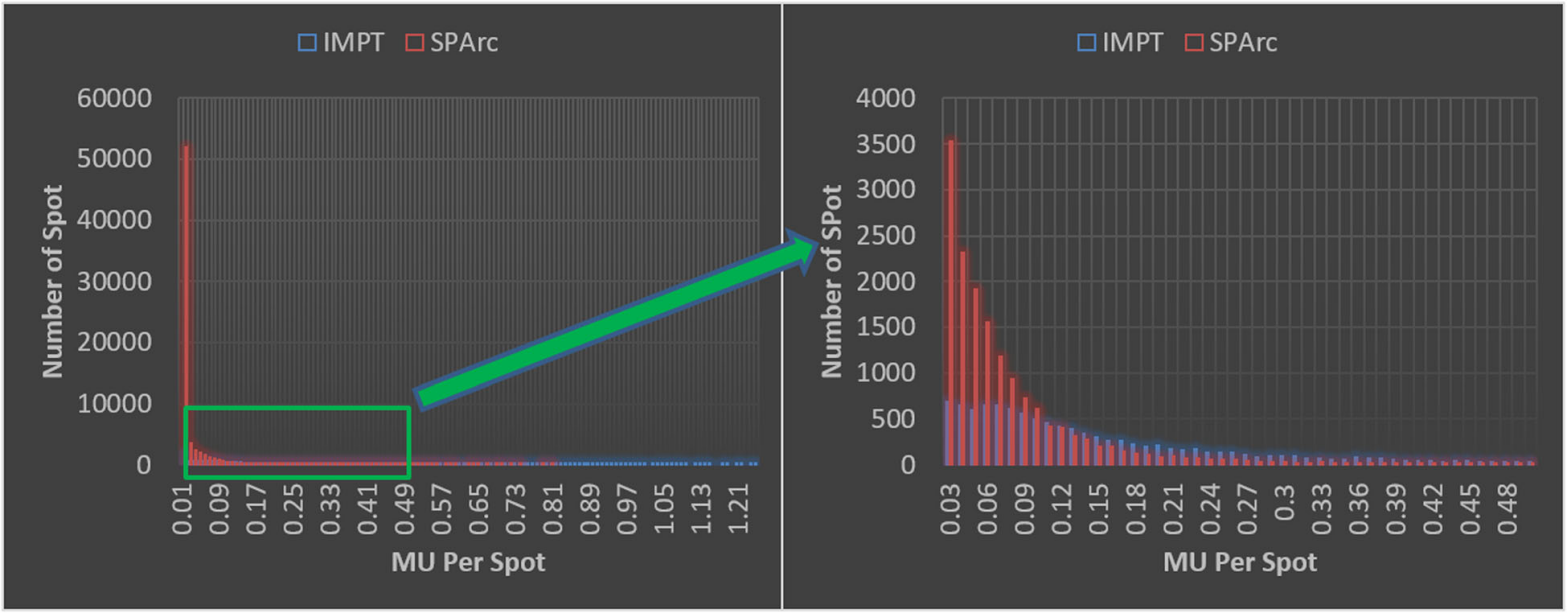
Spot vs MU weighting Histogram on an example patient case #8

At the current stage, the SPArc technique is still a concept that is under development towards the clinical product. One of the biggest challenges is the capability of delivering numerous spots with a significantly lower MU weighting compared to the traditional 3-field IMPT technique. Figure 5 showed an example of the spot weighting distribution comparison between the 3-field IMPT and SPArc plan for the HNC patient #8. The plan delivery accuracy and efficiency might be sensitive to the design of the beamline, ion chambers, and tolerance threshold of beam position and profile, which varies among different vendors or models of proton systems. The results from these QA experiments demonstrate the feasibility and compatibility of the SPArc delivery on an existing proton therapy system. However, we did observe one or two beams pauses/check on some SPArc plans treatment delivery, which was due to the noise of the ion chamber in delivering such highly modulated spots with low MU weightings. As a result, it is critical to understand the difference in the machine-specific limitations while generating the SPArc plan and arc delivery sequence. The next phase of development is to design a new control software and hardware which could deliver such proposed dynamic arc therapy in a safe, robust, accurate, and efficient way.

The use of RS in treating superficial cancer targets such as bilateral HNC complicates the clinical workflow in terms of the clearance check, especially near the shoulder region [12]. It also introduces secondary proton scattering from the RS, which increases spot size when entering the patient’s body and thus degrades the treatment plan quality due to the larger lateral penumbra [35–37]. There are numerous publications to address the clearance issues as well as proton beam modeling and dosimetric plan quality challenges of using RS [38, 39]. This study demonstrated that SPArc does not need to use RS while providing a superior dosimetric plan quality, simplifies the clinical workflow with a practically achievable treatment delivery time compared to the current standard-of-care IMPT.

Last but not the least, a more substantial volume of low dose bath is one of the biggest concerns in the proton arc therapy approach where the beam directions are coming from an arc(s) trajectory. The advantage of traditional proton beam therapy using limited beam angles can spare the normal tissue volume away from the target with almost zero doses. This feature is critical to the pediatric patients’ treatment, as most of them are expected to live much longer. Thus, it is crucial to reduce the chance of radiation-induced secondary malignancy by choosing the most appropriated beam angles [40, 41]. Our study showed that SPArc therapy has a higher V1Gy (*p* = 0.011) but a lower V3Gy (*p* = 0.008) in the treatment of bilateral HNC compared to the IMPT (Table 2). Although the 1Gy volume is higher, it is interesting to find that the total integral dose in bilateral HNC treatment is lower than IMPT which agrees with the findings in the lung and prostate studies as well [11, 20]. At the current stage, the clinical significance of low dose bath or integral dose in proton therapy is not well known yet, such concerns of radiation-induced secondary malignancy may limit the usage of proton arc technique in pediatric patients’ treatment. Based on this study, we are able to conclude that the significant dosimetric advantage of SPArc therapy is to offer a better dose conformity and a better sparing the high or median-dose volume which is critical to the disease sites where the OARs adjunct to the target volume such as HNC where the average age is between 50 and 70 years old [42]. Such a feature could allow proton beam therapy to reduce acute radiation toxicity furthermore.

## Conclusions

SPArc is a robust and delivery-efficient proton arc therapy technique that could potentially be implemented into routine clinical practice to further improve the treatment outcomes in HNC patients.

## Data Availability

All data generated or analyzed during this study are included in this published article. Additional information is available from the corresponding author on reasonable request.

## Abbreviations

3DCRT: 3-dimensional conformal radiation therapy
AUC: Areas Under the RVH Curve
CT: computed tomography
DVH: dose volume histogram
ELST: Energy Layer Switching Time
HI: Homogeneity Index
HNC: head and neck cancer
IMPT: Intensity Modulated Proton Therapy
MU: Monitor unit
NTCP: Normal tissue complication probability
OAR: Organs-at-risk
PBS: Pencil Beam Scanning
RBE: Relative biological effectiveness
RMSD: Root-mean-square dose
RS: Range shifter
RT: Radiotherapy
RTOG: Radiation Therapy Oncology Group
RVH: RMSD volume histograms
SPArc: Spot-scanning Proton Arc therapy
TPS: Treatment planning system
VMAT: Volumetric-modulated arc therapy
